# Myeloid-Derived Suppressor Cells Show Different Frequencies in Diabetics and Subjects with Arterial Hypertension

**DOI:** 10.1155/2019/1568457

**Published:** 2019-12-12

**Authors:** Julio C. Fernández-Ruiz, Julia C. Galindo-De Ávila, Margarita L. Martínez-Fierro, Idalia Garza-Veloz, Alberto R. Cervantes-Villagrana, Monica A. Valtierra-Alvarado, Carmen J. Serrano, Mariana H. García-Hernández, José A. Enciso-Moreno, Julio E. Castañeda-Delgado

**Affiliations:** ^1^Unidad de Investigación Biomédica de Zacatecas, Instituto Mexicano del Seguro Social, Zacatecas, Mexico; ^2^Centro de Investigación en Ciencias de la Salud y Biomedicina, Universidad Autónoma de San Luis Potosí, San Luis Potosí, Mexico; ^3^Maestría en Ciencias Biomédicas, Área de Ciencias de la Salud, Universidad Autónoma de Zacatecas, Zacatecas, Mexico; ^4^Laboratorio de Medicina Molecular, Unidad Académica de Medicina Humana y Ciencias de la Salud, Campus UAZ XXI, Universidad Autónoma de Zacatecas, Zacatecas, Mexico; ^5^Unidad Académica de Ciencias Químicas, Área de Ciencias de la Salud, Universidad Autónoma de Zacatecas, Zacatecas, Mexico; ^6^Cátedras CONACYT, Consejo Nacional de Ciencia y Tecnología, Mexico

## Abstract

Type 2 diabetes mellitus (DM2) is strongly associated with other comorbidities such as obesity, atherosclerosis, and hypertension. Obesity is associated with sustained low-grade inflammatory response due to the production of proinflammatory cytokines. This inflammatory process promotes the differentiation of some myeloid cells, including myeloid-derived suppressor cells (MDSCs). In this study, two groups of individuals were included: DM2 patients and non-DM2 individuals with similar characteristics. Immunolabeling of CD15+ CD14- and CD33+ HLA-DR-/low was performed from whole peripheral blood, and samples were analyzed by flow cytometry, and frequencies of MDSCs and the relationship of these with clinical variables, cytokine profile (measured by cytometric bead array), and anthropometric variables were analyzed. The frequency of CD33+ HLA-DR-/low MDSCs (that produce IL-10 and TGF-*β*, according to an intracellular detection) is higher in patients with DM2 (*P* < 0.05), and there is a positive correlation between the frequency of CD15+ CD14- and CD33+ HLA-DR-/low MDSC phenotypes. DM2 patients have an increased concentration of serum IL-5 (*P* < 0.05). Also, a negative correlation between the frequency of CD15+ CD14- MDSCs and LDL cholesterol was found. Our group of DM2 patients have an increased frequency of mononuclear MDSC CD33+ HLA-DR-/low that produce TGF-*β* and IL-10. These cytokines have been associated with immune modulation and reduced T cell responses. DM2 and non-DM2 subjects show a similar cytokine profile, but the DM2 patients have an increased concentration of IL-5.

## 1. Introduction

According to the American Diabetes Association (ADA), diabetes mellitus is a metabolic disease characterized by severe hyperglycemia due to defects in insulin secretion or the lack of proper action of this hormone in the target tissues. Type 2 diabetes mellitus (DM2) is the one that has the greatest impact worldwide, accounting for 90-95% of all reported cases of diabetes worldwide as reported by the ADA [1, 2]. The World Health Organization (WHO) estimated that 347 million cases of DM2 exist worldwide as of 2014 [3], and also, recent estimations suggest that DM2 will be the 7th cause of mortality by 2030 [4]. Data from the International Diabetes Federation suggest 4.9 million deaths associated with diabetes and its related complications [5]. In Mexico, the National Institute for Statistics and Geography (INEGI, acronym in Spanish) has reported that 70 out 1000 deaths were caused by diabetes and its complications causing a great economic burden to the national health institutions [6].

It has been described that the main factors associated with an increased risk of developing DM2 are obesity, unhealthy eating habits, sedentarism, advanced age, family history of diabetes, ethnicity, etc. [7–9]. The relationship between diabetes and obesity has been widely documented, and around 90% of diabetics are overweight or obese [10]. Obesity has also been associated with low-grade chronic inflammatory processes, and several cytokines such as tumor necrosis factor alpha (TNF-*α*) have been shown to be elevated in obese individuals due to an increased activity of adipose tissue-derived cytokine production and insulin resistance [11, 12]. Other bioactive molecules such as leptin, IL- (interleukin-) 6, resistin, and monocyte chemoattractant protein 1 (MCP-1) have been associated with insulin resistance [13–17].

Chronic inflammatory processes have been associated with alterations in leucocyte composition. In the seventies, a subset of myeloid cells with suppressor properties was described in patients with several types of cancer. These cells were at the moment described as “natural suppressor cells,” but the functional properties and markers of such cells have been recently described and named as myeloid-derived suppressor cells (MDSCs) [18–20]. These cells are a heterogeneous group of myeloid cells such as myeloid progenitors, immature myeloid cells, immature granulocytes, and immature dendritic cells. In humans, the main MDSCs have been described as immature monocytic and granulocytic cells, and several reports suggest an elevation in the frequency of MDSCs in some pathologic conditions, including cancer, sepsis, and acute and chronic inflammatory processes [19, 20]. Several phenotypes for these cells have been described such as CD15+ CD14- and also CD33+ HLA-DR-/low [19] to mention some [21]. It has been described that these cells have immune suppressive functions [22], and they are producers of interferon gamma (IFN-*γ*), IL-10, and transforming growth factor beta (TGF-*β*) [23].

As described previously, obesity is the biggest risk factor for developing DM2. This has also been associated with low-grade chronic inflammation with overproduction of TNF-*α*, IL-6, and IL-1*β* and other bioactive molecules in the adipose tissue such as granulocyte-macrophage colony-stimulating factor (GM-CSF), granulocyte colony-stimulating factor (G-CSF), macrophage colony-stimulating factor (M-CSF), vascular endothelial growth factor (VEGF), or IFN-*γ* [24–27]. Taking all the previously described data and the fact that diabetic individuals with DM2 have a higher predisposition to infection due to diminished immune responses [28–30], it becomes really important to understand if several cell populations that suppress the immune response such as the MDSCs are major conditioning factors that promote deficiencies in the development of several immune-mediated mechanisms in DM2 individuals [19]. The aim of the present paper was to compare the frequency of myeloid cells with the phenotypes CD15+ CD14- and CD33+ HLA-DR-/low producers of IL-10 and TGF-*β* in peripheral blood of patients with DM2 and non-DM2 subjects. The correlation between the MDSC immunophenotypes with common comorbidities to diabetics, laboratory, cytokine levels, cell suppressive function, and anthropometric data was also analyzed.

## 2. Material and Methods

### 2.1. Participants' Inclusion Criteria

DM2 patients of the study were recruited at the medical family care unit # 4 (Zacatecas, Mexico) according to approved protocols (R-2011-785-063); the visits were made between March 19 and May 19 in 2015. DM2 subjects (*n* = 23, age range of 35-62 years old) were invited to participate. DM2 subjects complied with the ADA criteria for DM2 diagnosis as follows: random glucose measurement of >120 mg/dl and/or glucose tolerance test > 200 mg/dl (for newly diagnosed individuals) and/or glycated hemoglobin (HbA1c) > 6.5%. Nondiabetic subjects (non-DM2) (*n* = 21) were negative for diabetes according to ADA criteria and were recruited at the same clinic with that from a similar age group. Clinical and laboratory data from all participants was collected from the routine follow-up at their clinic. Common comorbidities (reported by the treating physician) were also recovered from the medical records.

### 2.2. Ethics Statement

The present study was approved by the national commission on scientific investigation (CNIC) at the Mexican Institute for Social Security (IMSS) as well as the national ethics commission with registration number R-2011-785-063. All protocols were based on the International Declaration of Helsinki and in the principles of not malevolence, justice, and equality. All participants that agreed to participate signed an informed consent, and blood samples were drawn from only these individuals.

### 2.3. MDSC Phenotype and Flow Cytometry Analysis

Immunophenotypes were identified according to Ko et al. [31]. Briefly, blood samples were obtained from the participants by means of venipuncture. 4 ml of blood was collected. 100 *μ*l of whole blood was used with a lyse/wash protocol for staining. For the identification of the MDSC phenotypes, a combination of the following antibodies was used: CD14 PECy7 and CD15 PE-Cy5 (BD Biosciences, USA); CD33 APC and HLA-DR PerCP-Cy5.5 (BD Biosciences, USA). All antibodies were titrated before use. Immunostained samples were analyzed by flow cytometry in a FACSCanto II (Becton Dickinson, USA) with 488 and 633 laser lines and a standard configuration of 4-2 detectors. Data acquisition was performed by FACSDiva software v. 6.1 (BD Biosciences, USA), and 50000 events were acquired for each sample. The time parameter was used as quality control, observing stability between the performed analyses. Analysis of flow cytometry data was carried out in the FCS 3.0 files after export in FlowJo Software v. 8.7 (FlowJo LLC, USA).

### 2.4. Intracellular Staining of IL-10 and TGF-*β*

Blood samples were obtained from 4 DM2 patients and 4 nondiabetic participants by venipuncture. 100 *μ*l of whole blood was used with a lyse/wash protocol, and for cellular permeabilization, we used the BD Cytofix/Cytoperm™ Kit (BD Biosciences, USA) according to the manufacturer's instructions. Intracellular staining was performed immediately afterwards with the antibodies for TGF-*β* PE and IL-10 PE in separate tubes (BD, Biosciences, USA). Fluorescence minus one (FMO) was used as the control. Immunostained samples were analyzed by flow cytometry in a FACSCanto II (Becton Dickinson, USA), and analysis of flow cytometry data was carried out in the FCS 3.0 files after export in FlowJo Software v. 8.7 (FlowJo LLC, USA).

### 2.5. Cytokine Quantification by Cytometric Bead Array

The concentration of TNF-*α*, IFN-*γ*, IL-1*α*, IL-5, IL-10, IL-12p70, IL-17, and eotaxin was analyzed by cytometric bead array using the manufacturer's specifications (BD Biosciences, USA). Briefly, starting with cytokine standard preparation and cytokine capture bead mixture, 50 *μ*l serum samples from each individual and dilutions for the standard curve were placed in a multiscreen 1.2 *μ*m, 96-well filter plate (Merck Millipore, Germany), and the mixed capture beads and phycoerythrin detection reagent were added. After 3-hour incubation and repeated wash steps, the data was acquired in a FACSCanto II flow cytometer (Becton Dickinson, USA) and analyzed using FCAP Array v3.0 (BD Biosciences, USA) to convert fluorescent intensity values to concentrations using a standard curve.

### 2.6. Statistical Analysis

Normality of data was verified by means of a Kolmogorov-Smirnov test or a D'Agostino-Pearson normality test. Group comparisons for continuous quantitative variables were made with a Mann-Whitney *U* test for the nonparametric data or a Welch corrected *t*-test for normally distributed data. Categorical variables were analyzed with a Fisher exact test. A correlation analysis was made to explore the correlation of MDSC, laboratory, and anthropometric data by using a Spearman correlation (nonnormal distribution was verified). The two-tailed level of significance used for all analysis was *α* = 0.05. All statistical analysis was carried out in the GraphPad Prism software v6.0 (GraphPad Software, USA).

## 3. Results

### 3.1. Clinical Features of Patients with DM2 and Nondiabetic Controls

Clinical features, laboratory data, and anthropometric data were analyzed in order to determine whether differences were present due to heterogeneity of the population or whether differences in MDSC cells were associated with other parameters such as metabolic markers of disease or cardiovascular risk factors. As shown in Table 1, no major differences were identified in the groups in the variables such as age, sex, body mass index, waist to hip ratio, low-density lipoprotein cholesterol (cLDL), high-density lipoprotein cholesterol (cHDL), and total cholesterol. As expected, there were significant differences between DM2 subjects for both Hb1Ac% and glucose levels due to the inclusion criteria. No differences were identified either for markers associated with alterations in lipid metabolism or cardiovascular risk.

### 3.2. MDSCs with CD15+ CD14- and CD33+ HLA-DR-/Low Phenotypes Are Present in Both DM2 and Non-DM2 Subjects

**Figure 1:**
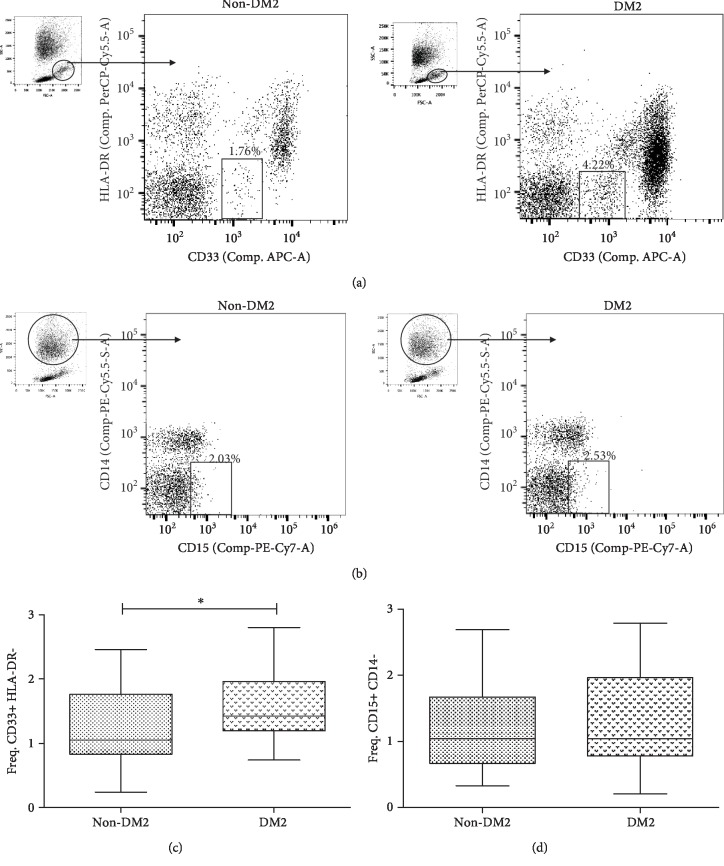
Gating strategy and frequencies of MDSCs in DM2. (a) Dotplot APC vs. PerCP-Cy5.5 shows PBMCs and the gate mark of the CD33+ HLA-DR-/low MDSCs. (b) Dotplot PE-Cy7 vs. PE-Cy5 shows PBMCs and PMNs and the gate mark of the CD15+ CD14- MDSCs. (c) Frequency of CD33+ HLA-DR-/low MDSCs, comparing the group of DM2 (*n* = 22) with the non-DM2 group (*n* = 21). The graph shows the median with interquartile ranges. (d) Frequency of CD15+ CD14- MDSCs, comparing the group of DM2 (*n* = 20) with the non-DM2 group (*n* = 21). The graph shows the mean and standard deviation. A *P* value < 0.05 was considered as statistically significant which was calculated using the statistical test of Mann-Whitney. Statistical analysis was performed with the GraphPad Prism® v5.0 software. Data were obtained in a BD FACSCanto II® Flow Cytometer and were analyzed with the software FlowJo® v10.0.

As mentioned above, several phenotypes of MDSCs have been identified and thoroughly characterized. The CD33+ HLA-DR-/low MDSCs are a subtype of myeloid cells reported by Ko et al. to have suppressive functions in renal cell carcinoma [31]. As shown in Figure 1(a), we identified such population with a similar gating strategy, and differences in both diabetics and nondiabetic subjects were also identified. For the CD15+ CD14- MDSC population (which has been described as another important subpopulation regulating in a negative manner the immune responses in some cancer patients) [31], samples of diabetics and nondiabetics show the presence of this population in low frequency (Figure 1(b)).

### 3.3. Patients with DM2 Have Different Frequencies of MDSCs

Given the suppressive role of MDSCs over other immune cells, one of the main goals of this study was to evaluate the frequency of MDSCs in patients with DM2 compared to non-DM2 subjects. For this purpose, the frequency of total cells was evaluated for both the CD33+ HLA-DR-/low and the CD15+ CD14- phenotypes. Significant differences were identified for the CD33+ HLA-DR-/low mononuclear MDSCs in patients with DM2 as compared to non-DM2 (*P* < 0.05, Figure 1(c)). For the CD15+ CD14- MDSC cell population, no differences between groups were found (*P* > 0.05, Figure 1(d)).

### 3.4. CD33+ HLA-DR-/Low MDSCs Produce TGF-*β* and IL-10

**Figure 2:**
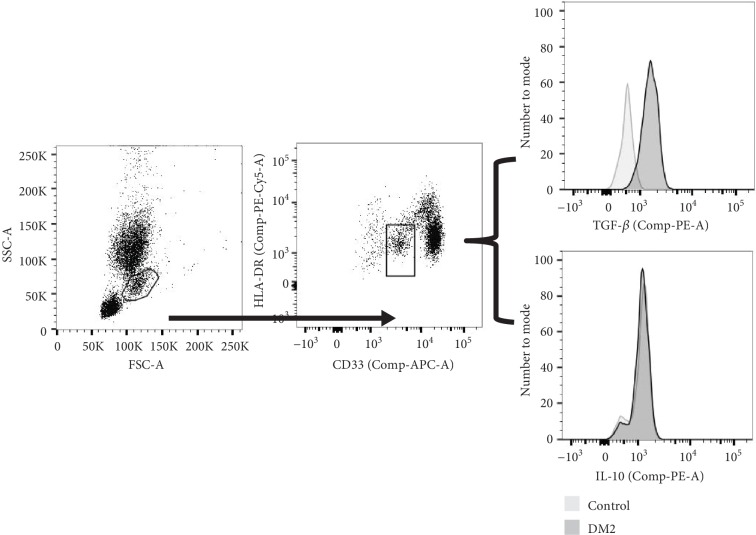
CD33+ HLA-DR-/low MDSCs produce IL10 and TGF-*β*. Representative data of the intracellular staining of IL-10 and TGF-*β* on CD33+ HLA-DR-/low MDSCs. Data were obtained in a BD FACSCanto II® Flow Cytometer and were analyzed with the software FlowJo® v10.0.

One of the major mechanisms by which MDSCs regulate the T cell-mediated immune response is by the production of IL-10 and TGF-*β*. Therefore, we evaluated the production of these cytokines in DM2 patients and controls. To demonstrate that the CD33+ HLA-DR-/low MDSCs are an immunoregulatory subset of cells, we did an intracellular staining of IL-10 and TGF-*β* and we found that this subpopulation actually produces these cytokines (Figure 2), suggesting that the immunosuppressive function of the MDSCs is also increased in the DM2 patients.

### 3.5. DM2 and Non-DM2 Subjects Show a Similar Cytokine Profile

Several reports suggest an association of increased frequencies of MDSCs and overproduction of inflammatory cytokines. Due to the elevation of the frequencies in the CD33+ HLA-DR-/low MDSCs in the diabetic group, we wondered whether this increased frequency of CD33+ HLA-DR-/low MDSCs could be related to cytokine overproduction. Therefore, several proinflammatory cytokines such as IL-1*α*, TNF-*α*, IFN-*γ*, IL-5, IL-12p70, and IL-17 and one anti-inflammatory IL-10 were quantified by cytometric bead array and analyzed through FCAP Array. The concentrations were classified for groups and compared between groups. Only significant differences were found in the comparison of IL-5 (*P* = 0.02); the other cytokines did not show any significant differences (Table 2). To corroborate, a correlation analysis was made between the concentrations of the cytokines and the frequencies of CD33+ HLA-DR-/low MDSCs. No correlation between these parameters was found (*P* > 0.05) (Supplementary [Supplementary-material supplementary-material-1]). Finally, the same correlation analysis was made; considering only the DM2 group, no significant correlations were identified either (Supplementary [Supplementary-material supplementary-material-1]).

### 3.6. CD33+ HLA-DR-/Low MDSC Increased Frequency Is Associated with Individuals with Hypertension

**Figure 3:**
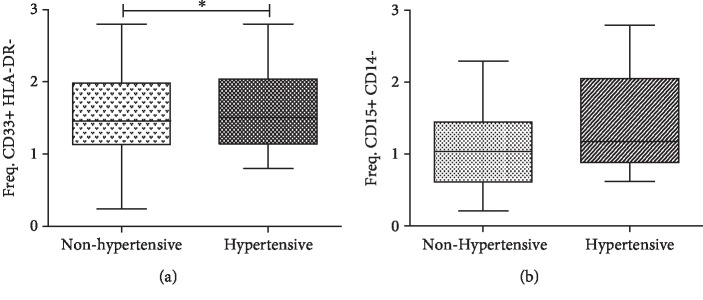
Frequency of CD33+ HLA-DR-/low and CD15+ CD14- MDSCs comparing if the subjects have hypertension in both groups (DM2 and non-DM2). (a) Frequency of CD33+ HLA-DR-/low MDSCs (hypertensive *n* = 16, nonhypertensive *n* = 27). (b) Frequency of CD15+ CD14- MDSCs (hypertensive *n* = 15, nonhypertensive *n* = 26). The graphs show the median and interquartile ranges. It was considered as statistically significant if *P* value < 0.05^∗^ which was calculated using the statistical *t*-test for unpaired samples. Statistical analysis was performed with the GraphPad Prism® v5.0 software.

It is known that some cell subtypes in the blood are associated with an increase in cardiovascular alterations and comorbidities, such as the case of T helper 17 (Th17) cells that respond to sodium increase in essential hypertension and have been recently linked to the pathogenesis of arterial hypertension [32, 33]. In order to identify if other variables were also associated with the increase in MDSCs, we evaluated if hypertension had any effect on the frequency of MDSCs. For this purpose, all subjects were polled for analysis and categorized according to having or not hypertension (reported in their clinical history) in two groups: hypertensive (which includes diabetic and nondiabetic) and nonhypertensive (which includes diabetic and nondiabetic). When the analysis was performed on these assumptions, we found a difference in the frequencies of CD33+ HLA-DR-/low MDSC phenotype (*P* < 0.05, Figure 3(a)) between hypertensive (mean = 1.620, SD = 0.602) and nonhypertensive (mean = 1.209, SD = 0.483) groups. These differences are not observed for the frequency of CD15+ CD14- MDSCs, although a slight increase is observed in the group that has hypertension (*P* > 0.05, Figure 3(b)).

### 3.7. MDSC Phenotypes Correlate with Cardiovascular Markers of Disease

**Figure 4:**
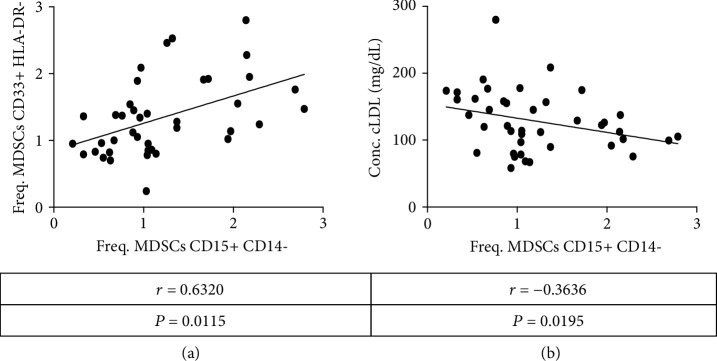
Correlation of MDSC frequency with cardiovascular markers. (a) Correlation between Freq. CD15+ CD14- and CD33+ HLA-DR-/low MDSCs. Data from 44 subjects, including groups in DM2 and nondiabetic controls. (b) Correlation between the concentration of LDL cholesterol and Freq. CD15+ CD14- MDSCs. Shown data are from 44 subjects (DM2 and nondiabetic controls). It was considered as statistically significant if value of *P* < 0.05. Statistical analysis was performed with the GraphPad Prism ® v5.0 software.

Given the previously shown results, MDSCs are elevated in the DM2 group and also in hypertensive individuals. To further clarify whether differences are associated with cardiovascular markers or diabetes markers, a correlation analysis was carried out. It was decided to perform the analysis for nonparametric data; of all the variables analyzed (age, BMI, WHR, glucose, Hb1ac%, cholesterol, cHDL, and cLDL), only the LDL cholesterol levels have a negatively statistically significant (*P* < 0.05) correlation with the frequency of CD15+ CD14- MDSCs (Figure 4(b)). Additionally, the frequency of CD33+ HLA-DR-/low and CD15+ CD14- MDSCs has a statistically significant correlation (*P* < 0.001, Figure 4(a)), and this correlation is maintained when only the DM2 group is analyzed (*P* = 0.029, Supplementary [Supplementary-material supplementary-material-1]), so this correlation is directly associated with DM2 condition. Data correlations with Spearman's rho value are summarized in Table 3.

## 4. Discussion

It is known that subjects with diabetes have an increased susceptibility to infectious diseases. For active tuberculosis infection, a risk of more than 5-fold has been reported in diabetics compared to nondiabetic subjects [34, 35]. Recent reports suggest that there are alterations in the immune cells such as neutrophil chemotactic and phagocytic functions that contribute to this increased susceptibility to infection in diabetics [36]; however, other cell populations might be associated with this phenomenon. MDSCs were linked in the last few years with increased susceptibility to infectious diseases; particularly, it was recently described that frequencies of these cells are elevated in patients with tuberculosis [37]. Recently, it has been shown that low-grade inflammation induced by obesity and infiltrating cytokine-producing adipose tissue immune cells is associated with several complications of diabetics such as insulin resistance [38]. Given that chronic inflammation is present in these individuals and due to the previously described reports that suggest that this might be associated with MDSC expansion, the present report evaluated whether or not diabetes-associated low-grade inflammatory processes might be associated with increased frequencies of MDSCs.

It has been described that there is a basal frequency of MDSCs in healthy individuals, although very small (around 1%) for both monocytic and polymorphonuclear MDSCs [31]; consistent with these findings, similar frequencies in our groups are reported. The physiological role of these cells is thought to be associated with immune regulation by several mechanisms, such as arginine depletion which causes a reduced activity of T cells and a decrease in CD62L in lymphocytes caused by ADAM metallopeptidase domain 17 (ADAM17), and through the expansion of regulatory T (Treg) cells by TGF-*β* secretion and IL-10 [20], we confirm that our population of CD33+ HLA-DR- mononuclear MDSCs produces IL-10 and TGF-*β* in both DM2 and nondiabetic donors.

A statistically significant rise in the frequency of CD33+ HLA-DR-/low MDSCs was found in the DM2 group; these findings are similar to those reported by Wang et al. [39] where they find an increased frequency of MDSC CD11b+ CD33+ and probe how these MDSCs inhibit T cell proliferation; although the phenotype is a little different (CD11b+ CD33+ vs. CDD33+ HLA-DR-/low), both correspond to a mononuclear immunophenotype; our findings reaffirm the importance that MDSCs could play in the pathophysiology of DM2 suppressing T cell response making patients susceptible to some infections and also provide a link to cardiovascular alterations in DM2 patients. The causes behind such increase could be associated with the production of TGF-*β* and IL-5. Several reports have described that the production of such cytokines in diabetics and obese people is increased and linked to insulin resistance [26]. Also, in diabetics, an increase in signaling pathways such as JNK and I*κ*B kinase beta (IKK*β*) has been described to be associated with insulin resistance, and these same signaling pathways are responsible of MDSC expansion in bone marrow [40–43]. Therefore, the low-grade inflammation process characteristic of diabetics could be associated with such observed expansion. Surprisingly, our results did not show differences in the cytokine profile, perhaps because all the patients were already under medical oversight and treatment. In our sample, there were no differences in the BMI (which is one of the variables associated with low-grade inflammation). Interestingly, the only cytokine that is elevated in our sample of DM2 was IL-5. This cytokine that has been reported to be increased in those patients with DM2 and tuberculosis [44], common comorbidity associated with immunodeficiency in DM2 patients; nevertheless, further investigation is needed to confirm the role of IL-5 in susceptibility to infections in DM2.

Systemic arterial hypertension (SAH) is a common comorbidity of DM2 patients, according to the National Health and Nutrition Survey (ENSANUT, acronym in Spanish) in 2016; in Mexico, patients with a previous diagnosis of diabetes have a prevalence of 57.6% with hypertension [45]. Therefore, when we analyzed according to SAH, a significant increase in MDSC frequency was found. Recent reports have documented that hypertension has an important inflammatory component particularly associated with Th17 cells that have a high response to salt concentrations [46]. Given that these cells are able to produce IL-6 and other cytokines [47] that are able to induce proliferation of MDSCs, our hypothesis is that such phenomena are intertwined, meaning that MDSC increased frequencies are probably a consequence of inflammatory processes from diabetes, obesity, and hypertension. This is further supported by our own results given that increased frequency of MDSC cells correlates between the two phenotypes; high levels of LDL cholesterol correlated negatively with CD15+ CD14- MDSCs, and this could be related to the generation of atheromatous plaques in blood vessels given that an increase of such inflammatory cells with phagocytic functions such as these is able to adhere to such plaques. These hypotheses need to be confirmed by experimental observations.

## 5. Conclusion

Our group of DM2 patients have an increased frequency of mononuclear MDSCs CD33+ HLA-DR-/low that produce TGF-*β* and IL-10. DM2 and non-DM2 subjects show a similar cytokine profile, but the DM2 patients have an increased concentration of IL-5. CD33+HLA-DR-/low MDSCs are associated with other complications of diabetics such as hypertension and cardiovascular markers of the disease (cLDL).

## Figures and Tables

**Table 1:** Clinical features of patients.

Variable	Group		*P* value
	Non-DM2 (*n* = 21)	DM2 (*n* = 23)	
Gender (female/male)	11/10	12/11	1.000^a^
Age (years)	43 (q1 = 42, q3 = 50)	50 (q1 = 40, q3 = 62)	0.264^b^
Years with diabetes		6.0 (q1 = 2, q3 = 14)	
BMI (kg/m^2^)	27.55 (q1 = 26.07, q3 = 30.22)	27.4 (q1 = 24.2, q3 = 28.7)	0.275^b^
Waist to hip ratio	0.94 (q1 = 0.91, q3 = 0.96)	0.93 (q1 = 0.89, q3 = 0.98)	0.913^b^
Glucose (mg/dl)	112 (q1 = 93, q3 = 117)	185.4 (q1 = 111, q3 = 233)	0.001^∗^^b^
Hb1Ac (%)	5.9 (q1 = 5.8, q3 = 6.2)	8.5 (q1 = 6.3, q3 = 10)	0.000^∗^^b^
Total cholesterol (mg/dl)	207 (q1 = 260, q3 = 231)	209.4 (q1 = 170, q3 = 230)	1.000^b^
cHDL (mg/dl)	48.3 (q1 = 40.9, q3 = 52.6)	49 (q1 = 38.2, q3 = 55.8)	0.869^b^
cLDL (mg/dl)	119.8 (q1 = 99.7, q3 = 156.3)	130.6 (q1 = 81.1, q3 = 160.9)	0.860^b^

^a^Fisher's exact test. ^b^Mann-Whitney *U* test. ^∗^*P* < 0.05.

**Table 2:** Comparison of serum cytokine concentrations among study groups^#^.

Variable	Non-DM2	DM2	*P* value
Eotaxin ($\bar{x}\pm \text{SD}$)	4475 pg/dl ± 1987	5338 pg/dl ± 2409	0.2127^a^

IL-1*α* (*m* ± IQR)	482.1 pg/dl q1 = 365.8, q3 = 665	415.1 pg/dl q1 = 323.1, q3 = 487.8	0.1741^b^

TNF-*α* (*m* ± IQR)	69.64 pg/dl q1 = 14.16, q3 = 149.4	42.75 pg/dl q1 = 34.94, q3 = 133.5	0.8591^b^

IFN-*γ* (*m* ± IQR)	642.8 pg/dl q1 = 397, q3 = 1534	400.1 pg/dl q1 = 83.98, q3 = 1252	0.4202^b^

IL-5 (*m* ± IQR)	2.850 pg/dl q1 = 0.4175, q3 = 15.89	38.4 pg/dl q1 = 4.42, q3 = 74.72	0.0200^∗^^b^

IL-10 (*m* ± IQR)	16.65 pg/dl q1 = 13.03, q3 = 32.66	26.68 pg/dl q1 = 11.49, q3 = 46.71	0.5652^b^

IL-12p70 (*m* ± IQR)	22.18 pg/dl q1 = 2.40, q3 = 60.23	22.65 pg/dl q1 = 16.11, q3 = 34.25	0.9004^b^

IL-17 (*m* ± IQR)	117.9 pg/dl q1 = 10.76, q3 = 298.4	93.97 pg/dl q1 = 65.10, q3 = 434.9	0.9999^b^

^#^Only the concentrations obtained above detection limits for each cytokine were considered for analysis. ^a^Student's *t*-test. ^b^Mann-Whitney *U* test. ^∗^*P* < 0.05.

**Table 3:** Correlation analysis of variables with the frequency of MDSCs^#^.

		Freq. MDSCs CD15+ CD14-	Freq. MDSCs CD33+ HLA-DR-
Age	Correlation coefficient	-0.083	-0.117
	Significance (two-tailed)	0.607	0.456
	*N*	41	43

Time of diagnosis (years)	Correlation coefficient	-0.080	0.039
	Significance (two-tailed)	0.744	0.869
	*N*	19	20

BMI (kg/m^2^)	Correlation coefficient	0.180	0.054
	Significance (two-tailed)	0.259	0.731
	*N*	41	43

Waist-hip ratio	Correlation coefficient	-0.259	-0.139
	Significance (two-tailed)	0.107	0.378
	*N*	40	42

Fasting glucose	Correlation coefficient	-0.097	0.252
	Significance (two-tailed)	0.547	0.103
	*N*	41	43

HbA1c (%)	Correlation coefficient	-0.040	0.255
	Significance (two-tailed)	0.802	0.100
	*N*	41	43

Total cholesterol (mg/dl)	Correlation coefficient	-0.284	-0.083
	Significance (two-tailed)	0.071	0.597
	*N*	41	43

cHDL (mg/dl)	Correlation coefficient	0.104	-0.044
	Significance (two-tailed)	0.518	0.779
	*N*	41	43

cLDL (mg/dl)	Correlation coefficient	-0.364^∗^	-0.110
	Significance (two-tailed)	0.019	0.482
	*N*	41	43

Triglycerides (mg/dl)	Correlation coefficient	-0.068	-0.068
	Significance (two-tailed)	0.675	0.665
	*N*	41	43

Freq. MDSCs CD15+ CD14-	Correlation coefficient	1.000	0.511^∗∗^
	Significance (two-tailed)		0.001
	*N*	41	40

Freq. MDSCs CD33+ HLA-DR-	Correlation coefficient	0.511^∗∗^	1.000
	Significance (two-tailed)	0.001	
	*N*	40	43

^#^Correlations were calculated with Spearman's rho. Differences in the sample size for each correlation may differ depending on the availability of the data for such patients or controls. ^∗^*P* < 0.05; ^∗∗^*P* < 0.01.

## Data Availability

The data used in this study are available from the corresponding author upon request.

## Supplementary Materials

Supplementary Materials Supplementary Table 1: correlation analysis of cytokine concentrations and MDSC frequency (CD33+ HLA-DR- phenotype). Supplementary Table 2: correlation analysis of cytokine concentrations and frequency of MDSCs CD15+ CD14- in the DM2 group. Supplementary Table 3: correlation analysis of variables with the frequency of MDSCs (only DM2 group).
