# Supplementary material for: Myeloid-Derived Suppressor Cells Show Different Frequencies in Diabetics and Subjects with Arterial Hypertension
Source: J Diabetes Res. 2019 Dec 12;2019:1568457. doi: 10.1155/2019/1568457 (PMC6930726; doi:10.1155/2019/1568457)
Supplement: Supplementary Materials — Supplementary Table 1: correlation analysis of cytokine concentrations and MDSC frequency (CD33+ HLA-DR- phenotype). Supplementary Table 2: correlation analysis of cytokine concentrations and frequency of MDSCs CD15+ CD14- in the DM2 group. Supplementary Table 3: correlation analysis of variables with the frequency of MDSCs (only DM2 group). [file 1568457.f1.docx]

| **Supplementary table 1:** Correlation analysis of cytokine concentrations and MDSCs frequency (CD33+ HLA-DR- phenotype). | | | | |
| --- | --- | --- | --- | --- |
|  | | | | |
| Variable |  | | Freq. MDSCs CD33+ HLA-DR- |  |
|  |  |  | |  |
| Eotaxin^a^ | Correlation coefficient  Significance (two tailed)  *N* | -0.032  0.8372  42 | |  |
| IL-1α^b^ | Correlation coefficient  Significance (two tailed)  *N* | 0.0918  0.5630  42 | |  |
| TNF-α^b^ | Correlation coefficient  Significance (two tailed)  *N* | 0.1264  0.6062  19 | |  |
| IFN-γ^b^ | Correlation coefficient  Significance (two tailed)  *N* | -0.0969  0.7664  12 | |  |
| IL-5^b^ | Correlation coefficient  Significance (two tailed)  *N* | 0.1617  0.5084  19 | |  |
| IL-10^b^ | Correlation coefficient  Significance (two tailed)  *N* | -0.2288  0.3318  20 | |  |
| IL-12p70^b^ | Correlation coefficient  Significance (two tailed)  *N* | -0.4909  0.1294  11 | |  |
| IL-17^b^ | Correlation coefficient  Significance (two tailed)  *N* | 0.0502  0.9039  9 | |  |

Correlation calculated with ^a^ Pearson´s rho and ^b^ Spearman´s rho. There was no significance p value for any of the data analized. Differences in the sample size for each correlation may differ depending on the availability of the data for such patients or controls.

| **Supplementary table 2:** Correlation analysis of cytokine concentrations and frequence of MDSCs CD15+ CD14- in DM2 group. | | | | |
| --- | --- | --- | --- | --- |
|  | | | | |
| Variable |  | | Freq. MDSCs CD15+ CD14- Diabetics group |  |
|  |  |  | |  |
| Eotaxin^a^ | Correlation coefficent  Significance (two tailed)  *N* | -0.0018  0.9935  21 | |  |
| IL-1α^b^ | Correlation coefficent  Significance (two tailed)  *N* | 0.2968  0.1913  21 | |  |
| TNF-α^b^ | Correlation coefficent  Significance (two tailed)  *N* | 0.5500  0.1328  9 | |  |
| IFN-γ^b^ | Correlation coefficent  Significance (two tailed)  *N* | 0.2000  0.9167  4 | |  |
| IL-5^b^ | Correlation coefficent  Significance (two tailed)  *N* | -0.0501  0.8754  11 | |  |
| IL-10^b^ | Correlation coefficent  Significance (two tailed)  *N* | -0.2242  0.5367  10 | |  |
| IL-12p70^b^ | Correlation coefficent  Significance (two tailed)  *N* | -0.5000  0.4500  5 | |  |
| IL-17^b^ | Correlation coefficent  Significance (two tailed)  *N* | 0.2029  0.7111  6 | |  |

Correlation calculated with ^a^ Pearson´s rho and ^b^ Spearman´s rho. There was no significance p value for any of the data analized. Differences in the sample size for each correlation may differ depending on the availability of the data for such patients or controls.

| **Supplementary Table 3.** Correlation analysis of variables with the frequence of MDSCs (Only DM2 group) ^a^. | | |  |
| --- | --- | --- | --- |
|  | Freq. MDSCs CD15+ CD14- | Freq. MDSCs CD33+ HLA-DR- | |
| Age | -0.30  0.900  20 | -0.100  0.656  22 | |
| Time of diagnosis (years) | -0.007  0.977  19 | 0.232  0.325  20 | |
| BMI | 0.125  0.600  20 | 0.042  0.853  22 | |
| waist-hip ratio | -0.412  0.071  20 | 0.055  0.809  22 | |
| Fasting glucose | 0.166  0.627  20 | -0.031  0.892  22 | |
| HbA1c (%) | 0.014  0.952  20 | -0.127  0.573  22 | |
| Total colesterol (mg/dL) | -0.220  0.352  20 | -0.094  0.676  22 | |
| HDLc (mg/dL) | 0.355  0.124  20 | 0092  0.682  22 | |
| LDLc (mg/dL) | -0.270  0.250  20 | -0.099  0.661  22 | |
| Triglycerides  (mg/dL) | -0.022  0.925  20 | 0.004  0.985  22 | |
| Freq. MDSCs CD15+ CD14- | 1.000  20 | 0.501*  0.029  19 | |
| Freq. MDSCs CD33+ HLA-DR- | 0.501*  0.029  19 | 1.000  22 | |
| a = Correlations were calculated with Spearman’s Rho. Differences in the sample size for each correlation may differ depending on the availability of the data for such patients or controls.  * = p < 0.05  ** = p < 0.01 | | |  |
